# Two new species and a new record of the *Encarsialongifasciata*-group (Hymenoptera, Aphelinidae) from Malaysia and China

**DOI:** 10.3897/BDJ.10.e91069

**Published:** 2022-09-26

**Authors:** Hui Geng, Cheng-De Li, Andrew Polaszek, Si-Zhu Liu

**Affiliations:** 1 Shangrao Normal University, Shangrao, China Shangrao Normal University Shangrao China; 2 Northeast Forestry University, Harbin, China Northeast Forestry University Harbin China; 3 Natural History Museum, London, United Kingdom Natural History Museum London United Kingdom; 4 Chongqing University of Posts and Telecommunications, Chongqing, China Chongqing University of Posts and Telecommunications Chongqing China

**Keywords:** Chalcidoidea, parasitoid, Southeast Asia, taxonomy

## Abstract

**Background:**

The genus *Encarsia* Förster, 1878, which is the largest genus of the family Aphelinidae, contains 453 valid species worldwide. Most species of *Encarsia* with known biology are primary endoparasitoids of Aleyrodidae and Diaspididae.

**New information:**

Species of the *Encarsialongifasciata*-group from Malaysia and China are reviewed. This is the first record of this group from Malaysia. Two new species, *E.borneensis* Geng & Li **sp. n.** and *E.pauroseta* Geng & Li **sp. n.**, are described and illustrated. *Encarsialongifasciata* is newly recorded from Malaysia (Borneo). An updated key to the *longifasciata*-group species (females) worldwide is provided.

## Introduction

So far, 108 and 11 species are described from China and Malaysia, respectively (Kresslein et al. 2020). There are more than 30 species groups proposed by authors in *Encarsia*, amongst them the *E.longifasciata*-group, together with *citrina*-, *parvella*-, *cubensis*- and *meghalayana*-group share the character of the fore wing having a clear asetose area around the stigmal vein. This group was defined, established and revised by Pedata and Polaszek (2003), with five species (*E.dewa*, *E.prinslooi*, *E.arabica*, *E.longifasciata* and *E.aleuroplati*). Schmidt and Polaszek (2007) described one additional species, *E.cassida*. Thus, currently six species are included in this group and all of them are known from the Old World tropics region. Here, we report on two new species of the *Encarsialongifasciata*-group and provide the first record of the species from this group from Malaysia.

## Materials and methods

Specimens were collected from Yunnan, China and Borneo, Malaysia using yellow pan traps. Specimens were dissected and mounted dorsally in Canada balsam on slides following the method of Noyes (1982). Morphologi­cal terminology and the measurement method follows Huang and Polaszek (1998), except metasoma is used for the petiole plus gaster.

Photographs were taken with a digital CCD camera attached to an Olympus BX51 compound microscope and most measurements were made from slide-mounted speci­mens using an eye-piece graticule. All the specimens listed below are deposited in the Northeast Forestry University, Harbin, China.

**The following abbreviations are used**:

**Fn** flagellar antennomeres.

**Tn** metasomal tergum.

**YPT** yellow pan trapping.

**NEFU** Northeast Forestry University, Harbin, China.

**NHMUK** Natural History Museum, London, UK.

## Taxon treatments

### Encarsia borneensis

Geng & Li sp. n.

4BD44D58-BFB8-599B-85B3-3EBC9C825603

#### Materials

- **Type status:** Holotype. **Occurrence:** individualCount: 1; sex: female; lifeStage: adult; **Location:** country: Malaysia; countryCode: MYS; stateProvince: Borneo; county: Sabah; municipality: Keningau; locality: Jungle Girl Camp; verbatimLatitude: 5°26'55.7" N; verbatimLongitude: 116°27'08.6" E; **Identification:** identificationID: E432; identifiedBy: Geng Hui; Li Cheng-De; **Event:** year: 2016; month: August; day: 21-25; **Record Level:** institutionCode: NEFU

#### Description

**Figure 1.**
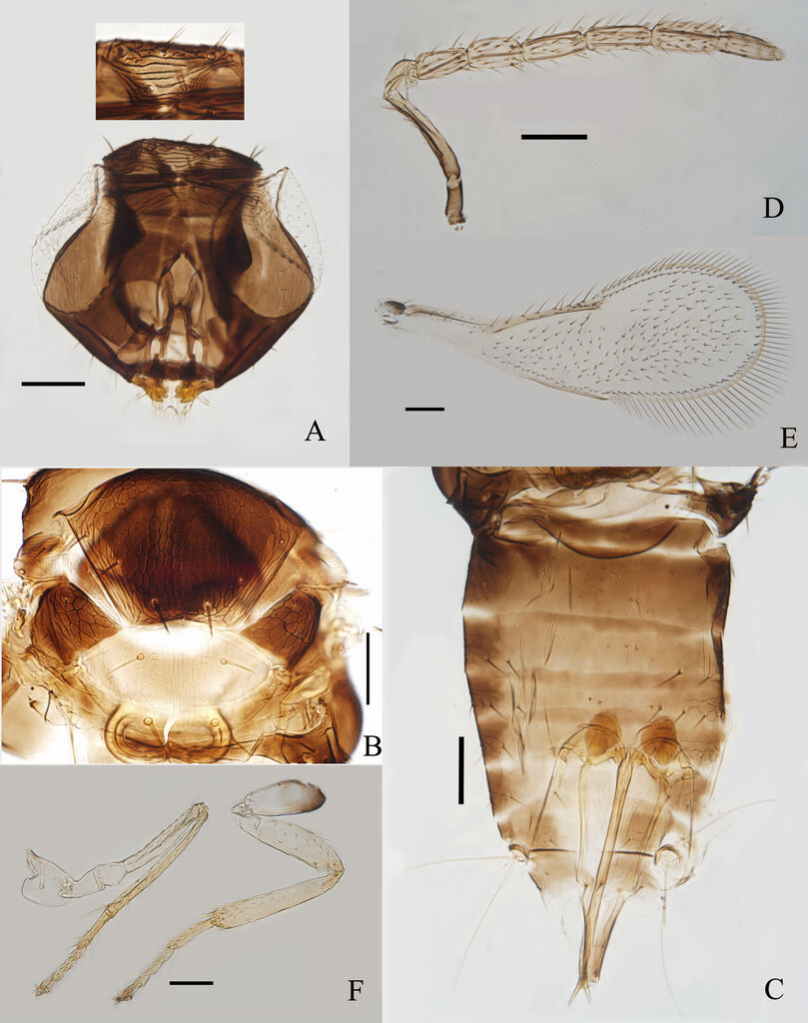
*Encarsiaborneensis* sp. nov. **A** head; **B** mesosoma antenna; **C** metasoma; **D** antenna; **E** fore wing; **F** mid- and hind leg. Scale bars = 50 μm.

Female (*Fig. 1*). Length, mesosoma plus metasoma, 0.57 mm. Head (Fig. 1A) dark brown with scrobes pale; eyes dark red, ocelli red. Mesosoma (Fig. 1B) with mid-lobe of mesoscutum dark brown, except sides and posterior margin light brown, axilla and propodeum dark brown, side lobe of mesoscutum brown-yellow with a dark patch anteromedially, scutellum pale yellow with posterior margin infuscate, metanotum pale brown-yellow. Metasoma (Fig. 1C) dark brown, except middle of T5-T6 and 3/4 apex of T7. Ovipositor yellow with third valvula brown. Antennae (Fig. 1D) yellow-brown with radicle and scape brown. Wings (Fig. 1E) hyaline, venation pale brown and tegula dark brown. Legs (Fig. 1F) pale yellow, except base of hind coxae brown.

Head (Fig. 1A) as wide as mesosoma. Maxillary and labial palps 1-segmented. Mandibles with three teeth. Eyes with fine and transpar­ent setae. Stemmaticum with transverse reticulate sculpture. Frontovertex with robust setae. Antennal formula 1,1,4,2 (Fig. 1D); F1 2× as long as wide, 1.5× as long as pedicel, as long as F2 and slightly shorter than F3, respectively. F2 and F3 1.83× and 2.5× as long as wide, respectively. Flagellum with the following numbers of longitudinal sensilla: F1:2, F2:2, F3:3, F4:4, F5:3, F6:3.

Mid-lobe of mesoscutum (Fig. 1B) with six setae, each side lobe with two setae. Axilla with one robust seta anteriorly. Mid-lobe of mesoscutum, axillae and scutellum with reticulate sculpture. Distance between placoid sensilla on scutellum 4.4× the maximum width of a sensillum. Distance between anterior pair of scutellar setae 1.31× as long as the distance between posterior pair. Fore wing (Fig. 1E) 2.74× as long as wide, with a clear asetose area around the stigmal vein and a wide asetose strip along posterior margin of wing disc; marginal fringe 0.37× as long as disc width, costal cell with nine setae in a row, basal cell with two setae, submarginal vein with two setae, marginal vein with five setae along anterior margin. Tarsal formula 5-5-5 (Fig. 1F). Mid-tibial spur 0.66× as long as correspond­ing basitarsus, the latter 0.35× as long as mid-tibia. Hind tibia 0.96× as long as mid-tibia.

Petiole smooth (Fig. 1C). T1–T5 laterally with scale like reticulation. T2– T7 with 2+2, 2+3, 1+1, 2+2, 1+2+1 and 4 setae, respectively. T7 1.29× as wide as long. Ovipositor exerted, apparently originating from level of T4, 1.19× as long as mid-tibia, 0.91× as long as mid-tibia and basitarsus combined. Third valvula 0.58× as long as second valvifer, 0.38× as long as ovipositor.

#### Diagnosis

Head and body largely dark brown, except scutellum and some pale spots on last three metasomal segments. Antennal F1 and F2 with longitudinal sensilla, F1 1.5× as long as pedicel. Mid-lobe of mesoscutum with three pairs of setae. Third valvulae brown and terminating in an abrupt angle.

#### Etymology

The specific name is derived from the collection locality name.

#### Taxon discussion

The new species is easy to distinguish from other species of this group by the combination of a dark metasoma, F1 with longitudinal sensilla and distinctly longer than pedicel and three pairs of setae on mid-lobe of mesoscutum.

### Encarsia pauroseta

Geng & Li sp. n.

8321763A-6DA0-5135-B0B6-AEE022374866

#### Materials

- **Type status:** Holotype. **Occurrence:** individualCount: 1; sex: female; lifeStage: adult; **Location:** country: China; countryCode: CN; stateProvince: Yunnan; county: Menghai; locality: Shuijingliangzi; **Identification:** identificationID: E172; identifiedBy: Geng Hui; Li Cheng-De; **Event:** year: 2014; month: February; day: 17-19; **Record Level:** institutionCode: NEFU

#### Description

**Figure 2.**
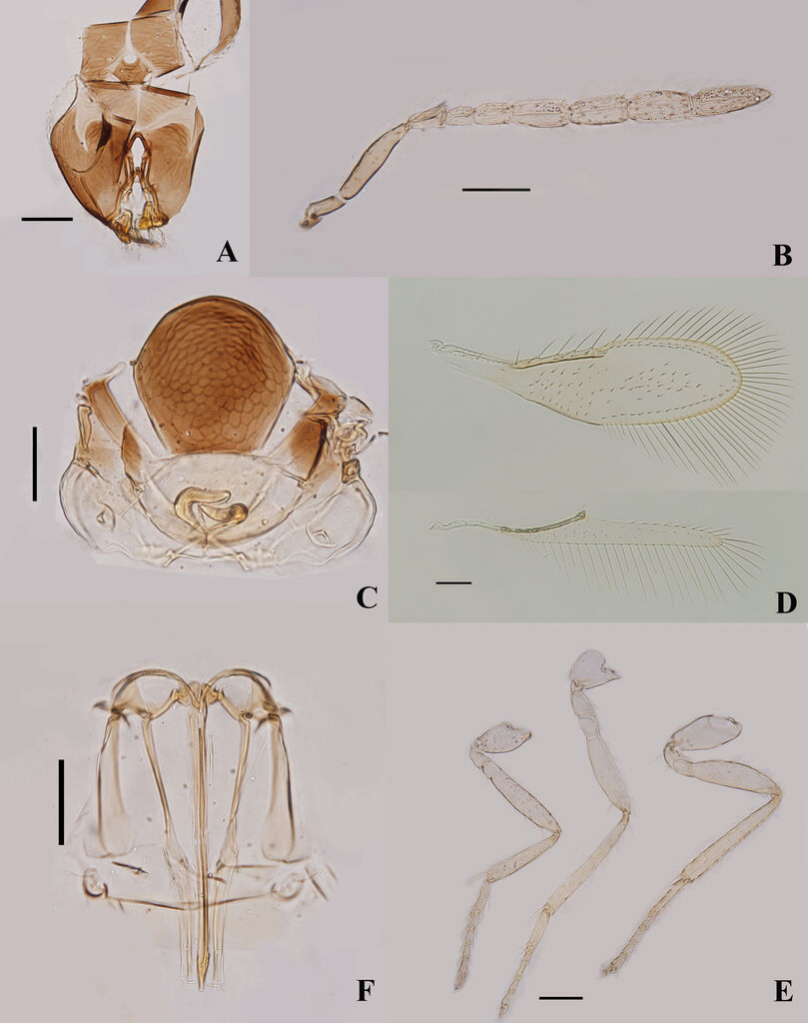
*Encarsiapauroseta* sp. nov. **A** head; **B** antenna; **C** metasoma; **D** wings; **E** legs; **F** ovipositor. Scale bars = 50 μm.

Female (Fig. 2). Holotype. Length, mesosoma plus metasoma, 0.44 mm. Head (Fig. 2A) dark brown with pale lines; clypeus and malar space dark brown. Mesosoma and metasoma yellow with pronotum, mid-lobe of mesoscutum (Fig. 2C) and axilla dark brown. Antennae (Fig. 2B) pale yellow with radicle and scape dark brown. Wings (Fig. 2D) hyaline. Legs (Fig. 2E) pale yellow. Apex of third valvula of ovipositor dark orange.

Head (Fig. 2A) as wide as mesosoma. Maxillary and labial palps 1-segmented. Stemmaticum with irregular reticulate sculpture. Antennal formula 1,1,4,2 (Fig. 2B); F1 1.33× as long as wide, 0.62× as long as pedicel, 0.67× as long as F2. F2 1.71× as long as wide. F1-F6 gradually longer. Flagellum with the following numbers of longitudinal sensilla: F1:0, F2:0, F3:3, F4:3, F5:3, F6:3.

Mid-lobe of mesoscutum (Fig. 2C) with two setae posteriorly, each side lobe with two setae. Axilla with one seta anteriorly. Mid-lobe of mesoscutum and scutellum with imbricate sculpture. Distance between placoid sensilla on scutellum 7× the maximum width of a sensillum. Distance between anterior pair of scutellar setae 0.9× as long as the distance between posterior pair. Fore wing (Fig. 2D) 3.6× as long as wide, sparsely setose, with a clear asetose area around stigmal vein and a wide asetose stripe along margin of disc; marginal fringe as long as width of disc; basal cell with one seta, submarginal vein with two short setae, marginal vein with four long setae along anterior margin. Hind wing 9.55× as long as wide, marginal fringe 2.25× as long as width of disc. Tarsal formula 5-5-5 (Fig. 2E). Mid-tibial spur 0.52× as long as correspond­ing basitarsus. Hind tibia 1.03× as long as mid-tibia.

Ovipositor (Fig. 2F) 1.23× as long as mid-tibia, 0.89× as long as mid-tibia and basitarsus combined. Third valvula 0.72× as long as second valvifer, 0.42× as long as ovipositor.

#### Diagnosis

Body largely yellow, except head, pronotum, mid-lobe of mesoscutum and axilla dark brown; mid-lobe of mesoscutum and scutellum with imbricate sculpture. Fore wing (Fig. 2D) 3.6× as long as wide, sparsely setose, with a clear asetose area around stigmal vein and a wide asetose stripe along margin of disc, marginal fringe as long as width of disc. Ovipositor 1.23× as long as mid-tibia; third valvula terminating in an abrupt angle.

#### Etymology

Ancient Greek παῦρος (paûros) small, little, referring to the sparse setation of the fore wing disc.

#### Taxon discussion

This species has unusual fore wings, the disc of which is very sparsely setose, with the maximum width as long as the maximum marginal fringe length. The remaining *E.longifasciata*-group species have the maximum marginal fringe length at most 0.7× as long as maximum disc width. This species appears to be similar to *E.dewa* Pedata & Polaszek having the mid-lobe with two setae, but can be distinguished from the latter by the following: marginal fringe of fore wing as long as width (vs. 0.65-0.7×), scutellum, tegula and metasoma totally pale yellow (vs. inverted triangular patch on scutellum, tegula and sides of metasoma brown), F2 0.67× as long as F3 and without longitudinal sensilla (vs. F2 subequal to slightly shorter than F3, with one longitudinal sensillum)，fore wing with a clear asetose area towards the apex (vs. fore wing with less asetose wing disc towards the apex).

### Encarsia longifasciata

Subba Rao, 1984

019B9B9D-C2E3-5325-82BC-E7350D131944

Encarsia longifasciata Subba Rao 1984: 260–261. Holotype ♀, India-Karnataka, (NHMUK, examined).
Encarsia longifasciata Subba Rao: Viggiani 1987: 35; Viggiani and Ren 1993: 223, 226; Huang and Polaszek 1998: 1906; Geng and Li 2016: 544.

#### Materials

- **Type status:** Other material. **Occurrence:** individualCount: 2; sex: female; lifeStage: adult; **Location:** country: Malaysia; countryCode: MYS; stateProvince: Borneo; county: Sabah; municipality: Keningau; locality: Jungle Girl Camp; verbatimLatitude: 5°26'55.7" N; verbatimLongitude: 116°27'08.6" E; **Identification:** identificationID: E448, E451; identifiedBy: Geng Hui; **Event:** year: 2016; month: August; day: 21-25; **Record Level:** institutionCode: NEFU

#### Notes

Two specimens agree with the descriptions given by Huang and Polaszek (1998) except narrower fore wing: marginal fringe of fore wing 0.68× as long as disc width and marginal vein with three to four setae along anterior margin. This is the first record of *E.longifasciata* from Malaysia.

## Identification Keys

### Key to species of *Encarsialongifasciata*-group (females) – update of the key in Pedata and Polaszek 2003

**Table:** 

1	Mid-lobe of mesoscutum with 3 pairs of setae	2
–	Mid-lobe of mesoscutum with 1-2 pairs of setae	3
2	Face and axillae dark brown; metasoma largely dark brown. F1 1.5× as long as pedicel and with two longitudinal sensilla	*E.borneensis* sp. n.
–	Face and axillae pale; metasoma largely pale, except T1-T4 laterally and T5 and T6 dark brown. F1 slightly shorter than pedicel and without longitudinal sensilla	*E.aleuroplati*
3	Mid-lobe of mesoscutum with 2 pairs of setae; fore wing more densely setose	4
–	Mid-lobe of mesoscutum with 1 pair of setae; fore wing more sparsely setose	5
4	Head dark brown; F1 distinctly shorter than F2	6
–	Head largely pale; F1 as long as F2	*E.prinslooi*
5	Scutellum almost totally pale	7
–	Scutellum largely pale with a dark triangular patch centrally	*E.dewa*
6	Anterior half of mid-lobe brown; basal cell with 4 setae; F1 0.64× as long as pedicel	*E.cassida*
–	Mid-lobe entirely brown, except sides and posterior margin light brown; basal cell with 1 seta; F1 subequal to slightly shorter than pedicel	*E.arabica*
7	Fore wing marginal fringe as long as disc width, with a clear asetose area around stigmal vein and a wide asetose strip along margin of disc; F2 1.71× as long as wide; third valvula 0.42× as long as ovipositor	*E.pauroseta* sp. n.
–	Fore wing marginal fringe 0.59× as disc width, with an asetose area around stigmal vein and a narrow asetose strip along distal and posterior margin of wing; F2 more than 2× as long as wide; third valvula 0.27-0.32× as long as ovipositor	*E.longifasciata*

## Supplementary Material

XML Treatment for Encarsia borneensis

XML Treatment for Encarsia pauroseta

XML Treatment for Encarsia longifasciata
